# Biological Characterization of Computationally Designed Analogs of peptide TVFTSWEEYLDWV (Pep2-8) with Increased PCSK9 Antagonistic Activity

**DOI:** 10.1038/s41598-018-35819-0

**Published:** 2019-02-20

**Authors:** Carmen Lammi, Jacopo Sgrignani, Anna Arnoldi, Giovanni Grazioso

**Affiliations:** 1 Dipartimento di Scienze Farmaceutiche, Università degli Studi di Milano, Via L. Mangiagalli 25, 20133 Milan, Italy; 2 Istituto di Ricerca in Biomedicina (IRB), Università della Svizzera Italiana (USI), Via V. Vela 6, CH-6500 Bellinzona, Switzerland

## Abstract

The inhibition of the PCSK9/LDLR protein-protein interaction (PPI) is a promising strategy for developing new hypocholesterolemic agents. Recently, new antibodies have been approved for therapy, but the high cost and low patients’ compliance stimulate the development of alternatives. Starting from the structural information available for the complex between PCSK9 and TVFTSWEEYLDWV (Pep2-8) peptide inhibitor and using computational methods, in this work we identified two Pep2-8 analogs as potential inhibitors of the PCSK9/LDLR PPI. Their biological characterization confirmed the theoretical outcomes. Remarkably, the treatment of HepG2 cells with these peptides increased the LDLR protein level on the cellular membrane, with activities that were 100 and 50 times better than the one of Pep2-8 tested at a 50 μM concentration. Moreover, they were 50 and 5 times more active than Pep2-8 in improving the functional ability of HepG2 cells to uptake extracellular LDL.

## introduction

Proprotein convertase subtilisin/kexin type 9 (PCSK9) is a serine protease belonging to the PC family, which is mainly expressed by the liver and small intestine^1^. Being a very promising target for the development of innovative treatments against hypercholesterolemia^2^, PCSK9 has attracted the attention of both the scientific community and pharmaceutical companies. In particular, large efforts have been devoted to the characterization of its physiological and pathophysiological roles. PCSK9 modulates low-density lipoprotein (LDL)-cholesterol (LDL-C) levels through its ability to mediate the LDL receptor (LDLR) protein degradation. The role of circulating PCSK9 in promoting hypercholesterolemia is strongly supported by preclinical experiments and clinical trials, where monoclonal antibodies (mAbs) directed against the LDLR binding site of PCSK9 efficiently reduce LDL-C levels^3^. In particular, experimental evidence is consistent with a mechanism in which the secreted form of PCSK9 directly binds the LDLR, inducing its degradation^4^. The LDLR binding to PCSK9 is stronger at acidic pH, suggesting that it occurs in the lysosomal/endosomal compartments^5^. Briefly, in the absence of PCSK9, the hepatic LDLR is shuttled back to the plasma membrane for degradation after cholesterol delivery to the lysosome, whereas the presence of PCSK9 prevents the LDLR shuttling and degradation^4^.

Since PCSK9 is a consolidated target for the management of plasma LDL-C levels, the main strategies for inhibiting PCSK9 have been based on the use of mAbs^6^, gene silencing compounds^7^, natural products, such as berberine^8^, or foods, such as lupins^9,10^, and peptidomimetics^11–13^. Currently, the most promising approach is represented by the use of mAbs: specifically, *alirocumab* and *evolocumab*, approved by the European Medicine Authority (EMA) in 2015, are already available on the market. They represent a successful strategy to inhibit PCSK9, but of course they are also very expensive and not always the patients’ compliance is satisfactory. These mAbs function by impairing the PCSK9-LDLR protein-protein interaction (PPI), thus validating this target for new drug discovery. To improve cost efficiency and patient compliance, the scientific community is looking for novel small molecules such as peptidomimetics able to impair this PPI.

Recently, innovative technological solutions for controlling peptide metabolism and advanced administration strategies^14–16^ have renewed the interest for novel inhibitory peptides. The seminal work of Shan *et al*. has provided the first evidence that this PPI can be successfully inhibited by a synthetic peptide mimicking the LDLR EGF-A domain^17^. Another study, carried out by us, has identified two peptides able to inhibit the PCSK9/LDLR PPI^11^: they derive from the hydrolysis of lupin protein and have the following sequences, LILPKHSDAD (P5) and GQEQSHQDEGVIVR (T9). The most active is P5, whose IC_50_ value is equal to 1.6 µM. Zhang and co-workers have identified some phage display-derived peptides as PCSK9 inhibitors, depositing in Protein Data Bank the crystal structures of PCSK9 in complex with TVFTSWEEYLDWV, a peptide known as Pep2-8^13^. More recently, the same authors described new analogs of Pep2-8, bearing extensions capable to confer binding affinities in the low micromolar range^18^.

**Figure 1:**
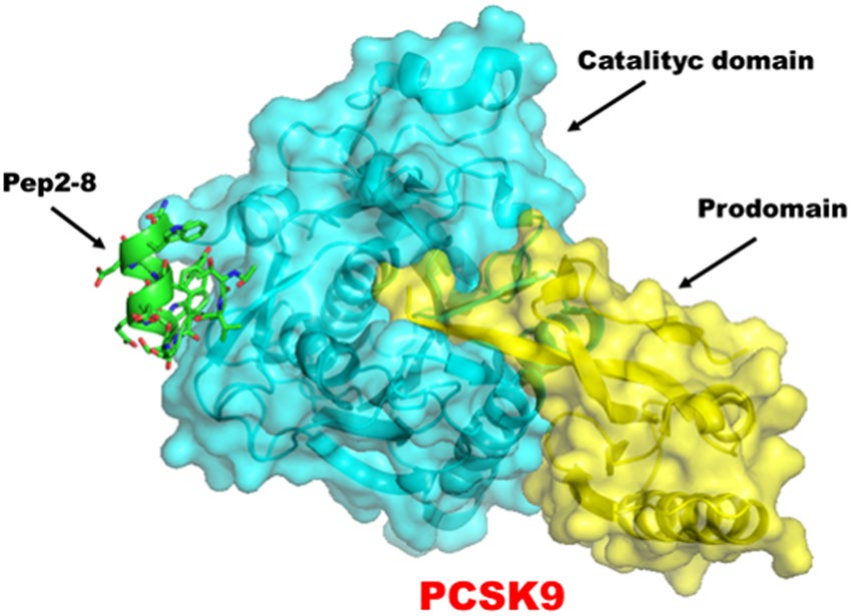
Representation of the PCSK9/Pep2-8 complex, as retrieved from Protein Data Bank, accession code 4NMX.

Starting from the X-ray crystal structure of the PCSK9/Pep2-8 complex (Fig. 1), in this work a computational alanine scanning mutagenesis of the ligand peptide was performed with the objective of recognizing the critical Pep2-8 residues (hotspots) mainly responsible for the interaction with PCSK9. A second objective was the identification of the positions inside the Pep2-8 sequence that, once modified, might increase the structural complementarity between Pep2-8 and PCSK9. The outcomes of the *in silico* research were completed and confirmed by *in vitro* biochemical assays and cellular investigations.

## Results

### PCSK9/Pep2-8 complex model

The 3D structure of the PCSK9/Pep2-8 complex was modeled and refined following the procedure described in the Experimental section. The starting pose of Pep2-8 was that found in the X-ray structure of the PCSK9/Pep2-8 complex (PDB accession code: 4NMX)^13^. The resulting complex model was equilibrated and optimized by means of 250 ns of molecular dynamics (MD) simulations^11^. The protein backbone was reasonably stable after the first 100 ns of MD simulations. However, the Calpha atoms of Pep2-8 showed a higher fluctuation than target, though within a strict range. The RMSD values vs. MD simulation time of Calpha atoms is reported in Fig. S1 (Supporting Information)”.

### Alanine scanning mutagenesis

PPIs are generally driven by residues located at the interface and those giving a major contribution to the binding energy of the interacting peptides are known as “hotspots”. Here, aiming to design new analogs with enhanced binding affinity to PCSK9, we applied alanine-scanning mutagenesis analysis to identify the Pep2-8 hotpots, as well as the role played by each residue constituting the primary structure of Pep2-8. Non “hotspots” residues were substituted by other amino acids, which might assure the best complementarity with the biological counterpart. This goal was achieved by target-based computational studies.

Alanine-scanning studies are usually carried out through the synthesis and biological evaluation of alanine single-point mutant peptides. Here, we preferred a different approach: performing molecular dynamics (MD) simulations on the complex containing the mutant peptides and applying the Molecular Mechanics-Generalized-Born Surface Area (MM-GBSA) approach it was possible to predict the binding free energies difference between the template and the alanine-mutant peptides with a good level of accuracy^19,20^.

In these calculations, the entropic contributions do not always improve the accuracy of the resulting binding free energy values^21–24^. Thus, also in order to avoid high demanding calculations, our MM-GBSA binding free energy predictions did not consider any entropic contribution, but resulted from the sum of the enthalpic and desolvation free energy items. For these reasons, our computations did not aim to reproduce experimental *K*_D_ values and should only to be considered an attempt to get a qualitative estimation of the peptide binding energy values (ΔG*), with the purpose of guiding the design of new Pep2-8 analogs^11,24,25^.

Our outcomes are collected in Table 1. In the next paragraphs, each residue composing the Pep2-8 sequence will be carefully analyzed in light of: (i) the interactions produced with PCSK9 in the X-ray crystal structure; (ii) the stability of those interactions over MD simulations, and (iii) the effects produced by the alanine mutation on the peptide binding free energy values. The detailed description of the MD simulations results is available as Supporting Material.

**Table 1:** Theoretical binding free energy values of the peptides under investigations, calculated by MM-GBSA approach (ΔG*, column 3).

Peptide	Sequence	Binding free energy [kcal/mol ± (Std. Err. of Mean)]	
		ΔG*	ΔΔG*
Pep2-8	**Ac-TVFTSWEEYLDWV-NH**_**2**_	−32.8 ± 0.9	0
[T1A]Pep2-8	**Ac**-**A**VFTSWEEYLDWV-**NH**_**2**_	−31.6 ± 0.5	+1.2
[V2A]Pep2-8	**Ac**-T**A**FTSWEEYLDWV-**NH**_**2**_	−31.7 ± 0.5	+1.1
[F3A]Pep2-8	**Ac**-TV**A**TSWEEYLDWV-**NH**_**2**_	−25.7 ± 0.4	+7.1
[T4A]Pep2-8	**Ac**-TVF**A**SWEEYLDWV-**NH**_**2**_	−34.2 ± 0.3	−1.4
[S5A]Pep2-8	**Ac**-TVFT**A**WEEYLDWV-**NH**_**2**_	−31.4 ± 0.4	+1.4
[W6A]Pep2-8	**Ac**-TVFTS**A**EEYLDWV-**NH**_**2**_	−28.6 ± 0.5	+4.2
[E7A]Pep2-8	**Ac**-TVFTSW**A**EYLDWV-**NH**_**2**_	−30.3 ± 0.4	+2.5
[E8A]Pep2-8	**Ac**-TVFTSWE**A**YLDWV-**NH**_**2**_	−31.4 ± 0.5	+1.4
[Y9A]Pep2-8	**Ac**-TVFTSWEE**A**LDWV-**NH**_**2**_	−37.4 ± 0.5	−4.6
[L10A]Pep2-8	**Ac**-TVFTSWEEY**A**DWV-**NH**_**2**_	−31.6 ± 0.5	+1.2
[D11A]Pep2-8	**Ac**-TVFTSWEEYL**A**WV-**NH**_**2**_	−28.9 ± 0.3	+3.9
[W12A]Pep2-8	**Ac**-TVFTSWEEYLD**A**V-**NH**_**2**_	−35.5 ± 0.4	−2.7
[V13A]Pep2-8	**Ac**-TVFTSWEEYLDW**A**-**NH**_**2**_	−32.5 ± 0.4	−0.3

ΔΔG* values (column 4) represent the difference between the theoretical binding free energy value of Pep2-8 and those calculated for the alanine-mutants. A positive value suggests a lower affinity between the peptide and PCSK9. The N-terminus and C-terminus of the peptides were protected by acetyl and amino groups, respectively.

### Identification of the hotspots

Table 1 shows that F3A and W6A mutations markedly reduce peptide/PCSK9 contacts. In fact, the F3A and W6A point mutations led to peptides with ΔG* values 7.1 and 4.2 kcal/mol higher than the value calculated for Pep2-8. This clearly suggests that F3 and W6 could be considered PCSK9/Pep2-8 PPI hotspots.

More in depth, in the crystal pose, the phenyl ring of F3 is packed against the side chains of Pep2-8W12 and Pep2-8Y9 and this interaction is stable over MD simulations. Conversely, MD simulations on the PCSK9/[F3A]Pep2-8 complex showed a huge conformational instability of the mutant peptide within PCSK9. In fact, the lacking of the phenyl ring into the *core* of the small peptide led to huge conformational mobility of the peptide N-terminal end. Finally, when the peptide/PCSK9 complex reached the geometrical stability, the acetyl group (capping the peptide on T1) substituted the benzyl group of Pep2-8F3 in the contacts produced with the side chains of W12 and Y9 and with PCSK9. Additionally, a high fluctuation of the hydrogen bond (Hb) between the NH group of Pep2-8F3 and the carbonyl group of PCSK9-F379 was noted (see Supporting Material for details).

Similarly, position 6 could be considered a hotspot because, during MD simulations, the indole ring of W6 was (i) in contact with the phenol ring of Pep2-8Y9 and (ii) inserted into the PCSK9 cavity sized by D238, F379, I369, P155 and. The MD trajectory of the PCSK9/[W6A]Pep2-8 complex showed that, at the end of the simulations, the α-helix of the mutant peptide was shifted in the direction of PCSK9-T377 and the PCSK9-C375/C378 disulfide bridge. Moreover, the side chain of Pep2-8Y9 replaced the W6 indole ring. Consequently, these alterations in peptide binding mode resulted in a higher predicted ΔG* value.

### Gainful mutations

**Figure 2:**
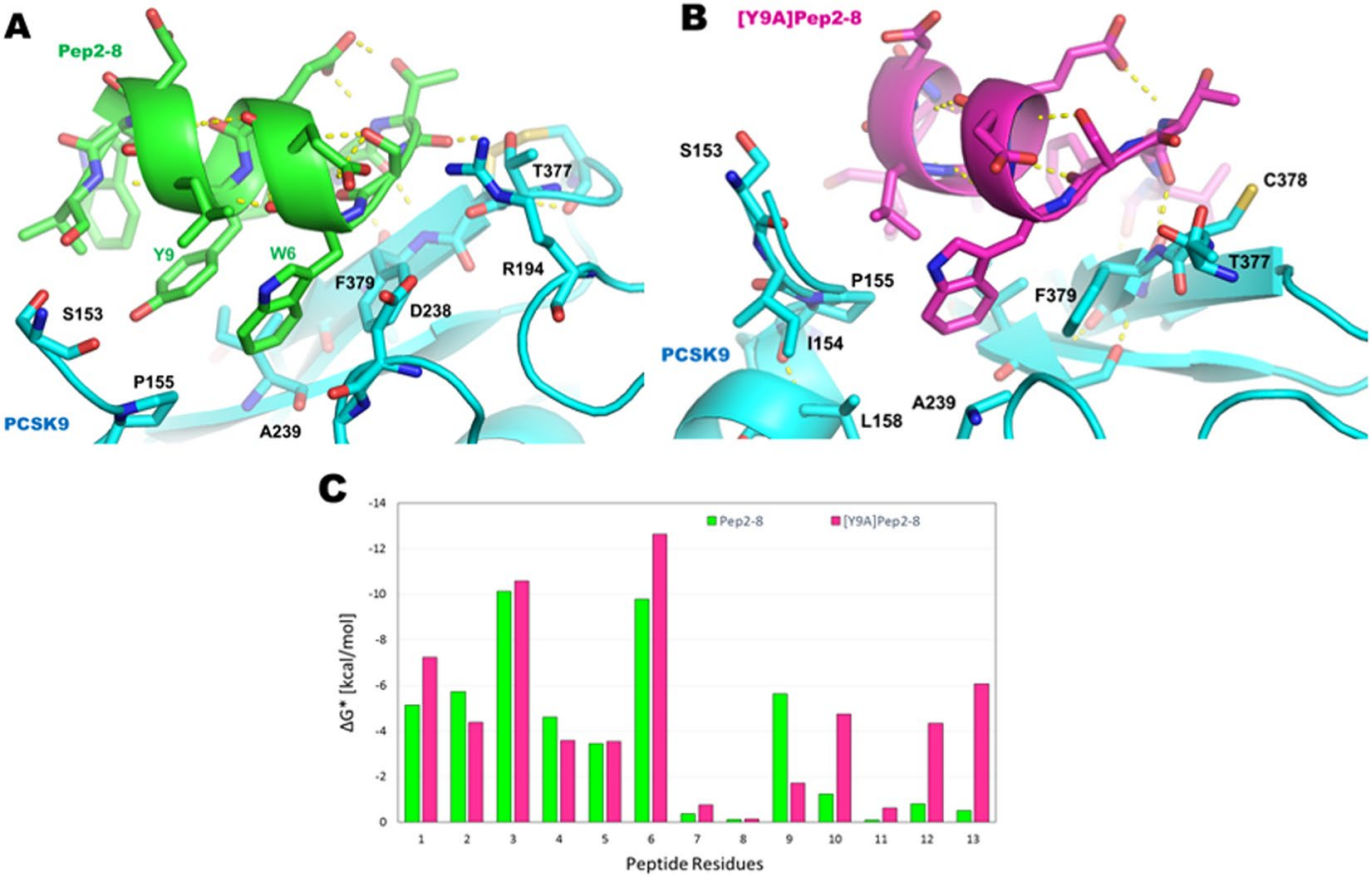
Proposed binding modes of Pep2-8 (panel **A**) and [Y9A]Pep2-8 (panel **B**) within PCSK9, at the end of MD simulations. (**C**) Histogram showing the calculated per-residue free decomposition energy using the MM-GBSA approach.

Negative ΔΔG* values of mutant peptides suggest an increased peptide affinity to the PCSK9 binding site. The alanine mutation of Pep2-8 in position 9 led to the greatest ΔG* improvement. The visual inspection of the MD trajectory showed some improved [Y9A]Pep2-8/PCSK9 contacts: (1) the methyl group of the alanine substituting Pep2-8Y9 was oriented toward the side chain of PCSK9-I369; (2) the side chain of Pep2-8L10 replaced the contact of the phenol ring of Pep2-8Y9 in the pocket shaped by PCSK9-P155 and -I369; (3) the side chain of Pep2-8V3 established van der Wall contacts with PCSK9-P155, Pep2-8W4 and PCSK9-D367; (4) the side chain of Pep2-8W12 left its contact with the side chain of PCSK9-D367; (5) the side chain of Pep2-8F3 made contact with the side chain of PCSK9-I369, as well as with the isopropyl moiety of Pep2-8V13 (Fig. 2B). MM-GBSA calculations corroborate these theoretical observations. In fact, the ΔG* value predicted for [Y9A]Pep2-8 was significantly lower than that of Pep2-8 (−37.4 kcal/mol *versus* −32.8 kcal/mol), a fact that strongly suggests that the removal of the phenol ring in position 9 of Pep2-8 may lead to a better ligand-target reciprocal adaptation. Additionally, a per-residue decomposition of the MM-GBSA data was performed in order to define the contribution of each residue in [Y9A]Pep2-8. These calculations showed that residues 6, 10, 12 and 13 of the mutant peptide created stronger interactions with PCSK9 (magenta bars in Fig. 2B).

### Neutral mutations

When mutated into alanine, residue in positions 2 led to a peptide with ΔG* value slightly higher than Pep2-8, as confirmed by Zhang *et al*.^18^, whereas mutation [V2A]Pep2-8 showed low affinity on PCSK9. Similarly, the mutation of residues in positions 1, 5, 7, 8, 10, and 13 of Pep2-8 influenced only marginally the theoretical binding affinity, since the predicted ΔG* values were very similar to the reference value (differences smaller than 2 kcal/mol). Conversely, the ΔG* value predicted for [D11A]Pep2-8 was 3.9 kcal/mol higher than the reference value. This data appears anomalous since the side chain of D11 does not play any crucial role in the Pep2-8/PCSK9 PPI, being located on the external surface of the complex. Interestingly, the T4A and W12A mutations led to peptides showing a little improvement in the theoretical binding affinity. In fact, the side chains of both residues are in close contact with PCSK9, therefore these sites represent the most interesting positions in which the sequence of Pep2-8 could be altered aiming to design new Pep2-8 analogs with improved biological activity.

### Design of new Pep2-8 analogs

**Figure 3:**
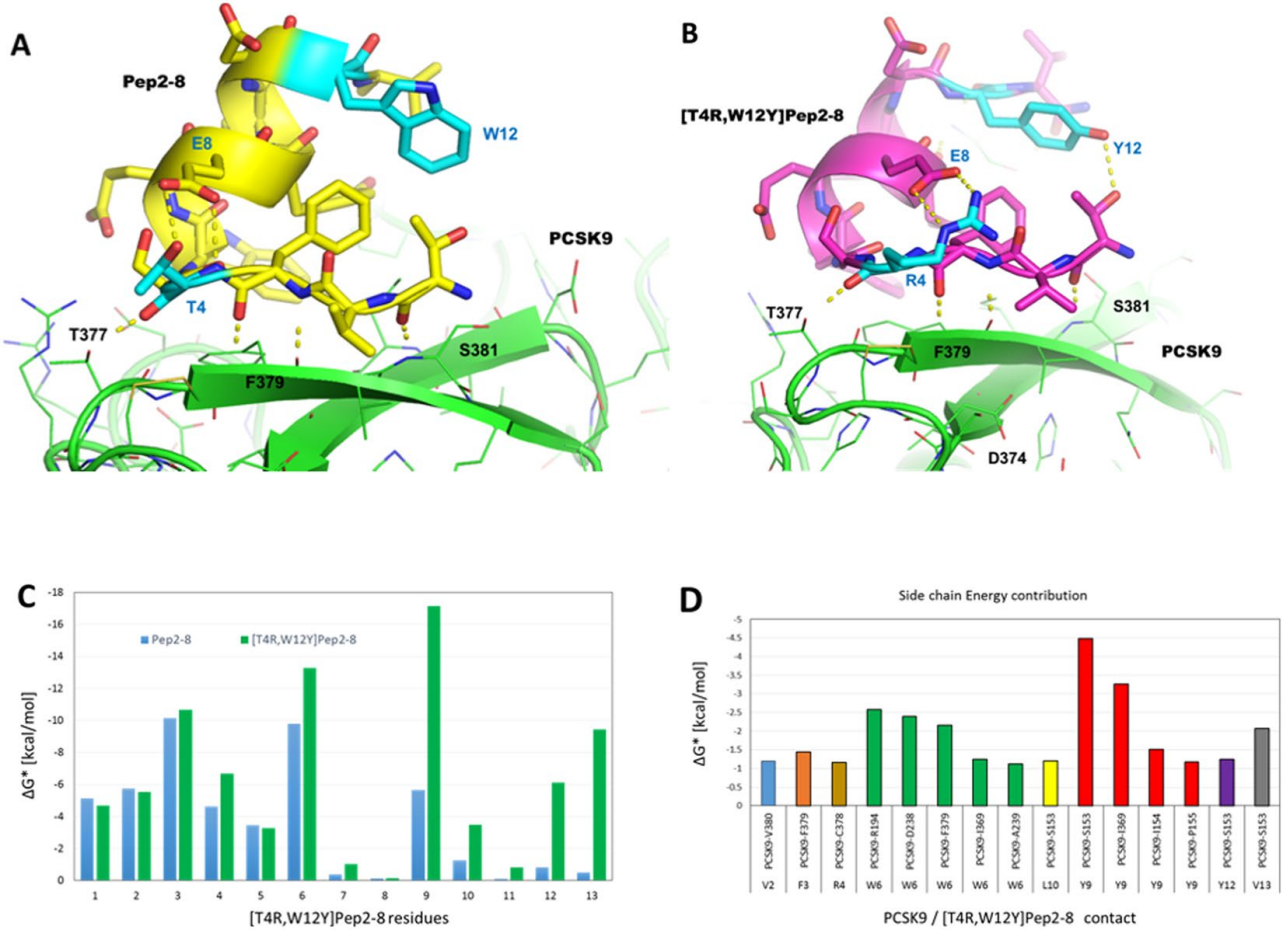
(**A**) Binding mode of Pep2-8 (yellow sticks) in the conformation equilibrated by MD simulations. (**B**) Hypothetical binding mode of [T4R,W12Y]Pep2-8 (magenta sticks), after 150 ns of MD simulations. To highlight the mutation sites, residues in position 4 and 12 are represented as cyan sticks. PCSK9 is represented as green model and dotted yellow lines depict the Hb network. (**C**,**D**) Histograms showing the calculated per-residue free decomposition energy using the MMGBSA approach.

In agreement with previous considerations, position 4 and 12 are the most suitable sites for Pep2-8 mutation. Consequently, all 400 possible analogs were designed and their affinities for PCSK9 were evaluated by computational analysis. Finally, the complexes between PCSK9 and the three top ranked peptides (i.e. those showing the lowest ΔAffinity values, Table 2) were simulated by MD and the affinity estimated again by MM-GBSA calculations (Table 2). Interestingly, all three analogs showed comparable ΔG* values, which were significantly lower than that calculated for Pep2-8 (Table 2). Among them, [T4R,W12Y]Pep2-8 attained the most encouraging prediction (Fig. 3A,B). The improved ΔG* value of [T4R,W12Y]Pep2-8 was examined by MM-GBSA/energy decomposition calculations (Fig. 3C,D). The results showed that the residues mainly responsible for its increased affinity were those in position 6, 9, 12 and 13 (Fig. 3C). In particular, [T4R,W12Y]Pep2-8W6 creates closer contacts with a PCSK9 pocket-sized by residues R194, D238, F379, I369 and A239 (Fig. 3B). Similarly, [T4R,W12Y]Pep2-8Y9 interacts with a PCSK9 area shaped by S153, I369, I154 and P155. The C-terminal end of the mutant peptide creates hydrogen bonds with the N-terminal end of the enzyme, constituted by the PCSK9-S153 (Fig. 3B), as confirmed by the visual inspection of the MD trajectory.

**Table 2:** Theoretical binding affinity values of the new designed peptides (column 1–2), calculated by Prime software (www.Schrodinger.com, ΔAffinity, column 3) and by standard MD/MM-GBSA calculations (ΔG* values, column 4).

Peptides	Sequence	ΔAffinity [kcal/mol]	ΔG* value [kcal/mol ± (Std. Err. of Mean)]
**Pep2-8**	**Ac-TVFTSWEEYLDWV-NH**_**2**_	—	−32.8 ± 0.9
[Y9A]Pep2-8	**Ac-**TVFTSWEE**A**LDWV**-NH**_**2**_	—	−37.4 ± 0.5
[T4R,W12Y]Pep2-8	**Ac-**TVF**R**SWEEYLD**Y**V**-NH**_**2**_	−12.9	−39.8 ± 0.4
[T4Y,W12Y]Pep2-8	**Ac-**TVF**Y**SWEEYLD**Y**V**-NH**_**2**_	−9.7	−40.4 ± 0.4
[T4W,W12Y]Pep2-8	**Ac-**TVF**W**SWEEYLD**Y**V**-NH**_**2**_	−9.6	−38.4 ± 0.7

See Experimental section for details.

Accordingly with these outcomes, the most interesting peptide in the alanine-scanning mutational analysis, i.e. [T4R,W12Y]Pep2-8 and [Y9A]Pep2-8, were synthetized and submitted to experimental investigations.

### Engineered peptides inhibit the PCSK9-LDLR binding

**Figure 4:**
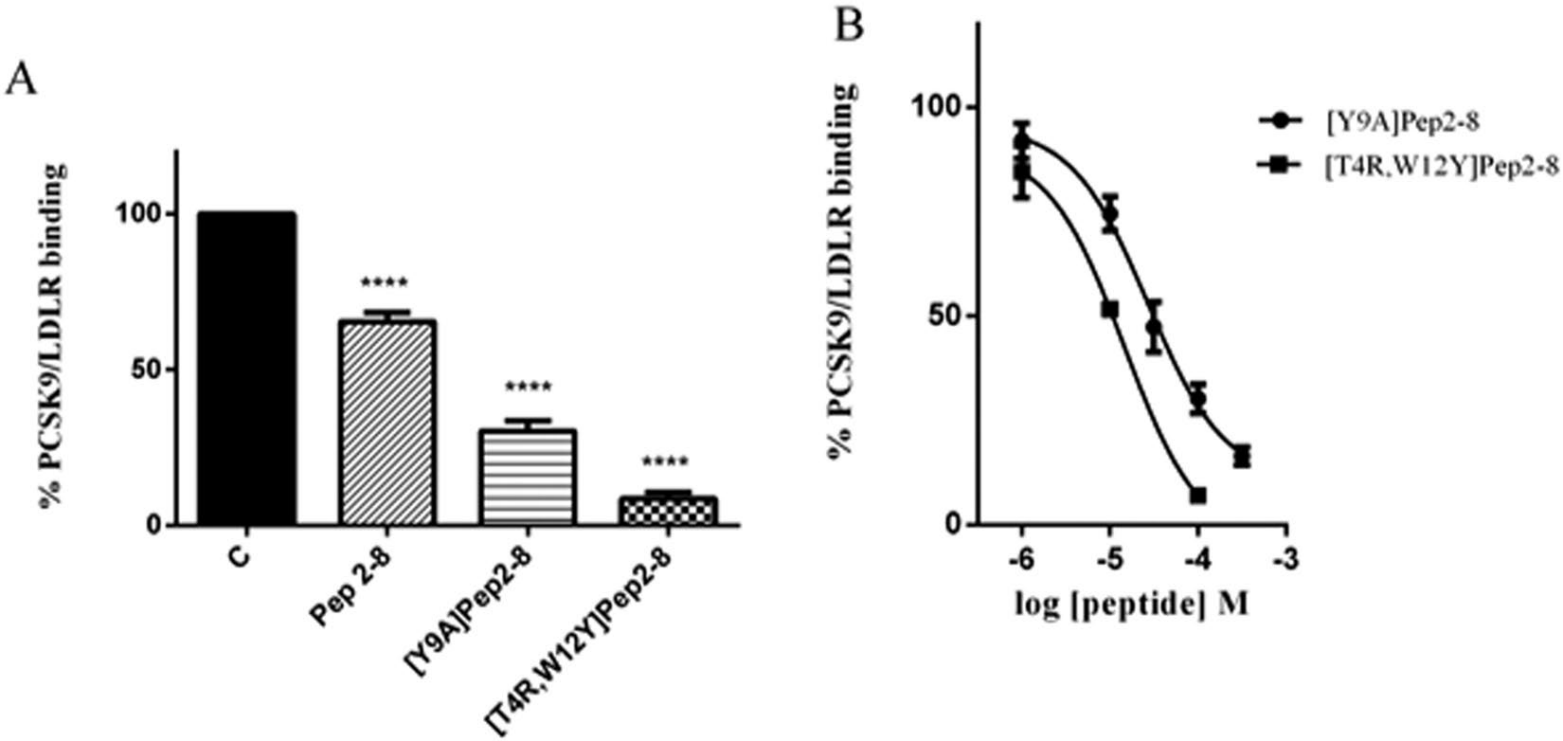
Pep2-8 analogs inhibit the PCSK9-LDLR binding. Inhibitory effects of (**A**) [Y9A]Pep2-8 and [T4R,W12Y]Pep2-8 (100 µM) on the PCSK9-LDLR PPI *in vitro*. Experiments have been performed using Pep2-8 as reference compound at the fixed concentration of 100 µM. (**B**) [Y9A]Pep2-8 and [T4R,W12Y]Pep2-8 showed a concentration-response behavior, with IC_50_ equal to 27.12 ± 1.2 µM and 14.50 ± 1.3 μM, respectively. Data points represent averages ± s.d. of three independent experiments in duplicate. (****) *P* < 0.00001. C represents the control without any treatment.

Initially, *in vitro* binding experiments were performed in order to evaluate the ability of peptides [Y9A]Pep2-8 and [T4R,W12Y]Pep2-8 to inhibit the PCSK9/LDLR PPI. As a first screening, the activities of the new peptides and reference peptide Pep2-8 were compared at the fixed concentration of 100 µM. Indeed, the new analogs resulted to be more active than the reference peptide, since Pep2-8 impaired the PCSK9-LDLR binding by −36.5% *versus* the control, whereas [Y9A]Pep2-8 and [T4R,W12Y]Pep2-8 by −69.8% and −93.0%, respectively (Fig. 4B). Subsequently, dose-response curves were built for the new peptides: the IC_50_ value of [Y9A]Pep2-8 was equal to 27.12 ± 1.2 µM and that of [T4R,W12Y]Pep2-8 equal to 14.50 ± 1.3 µM (Fig. 4B). The theoretical predictions appeared therefore well confirmed by the experimental assays.

### PCSK9 inhibition by Pep2-8 analogs increased the LDLR protein levels on HepG2 cellular membrane

**Figure 5:**
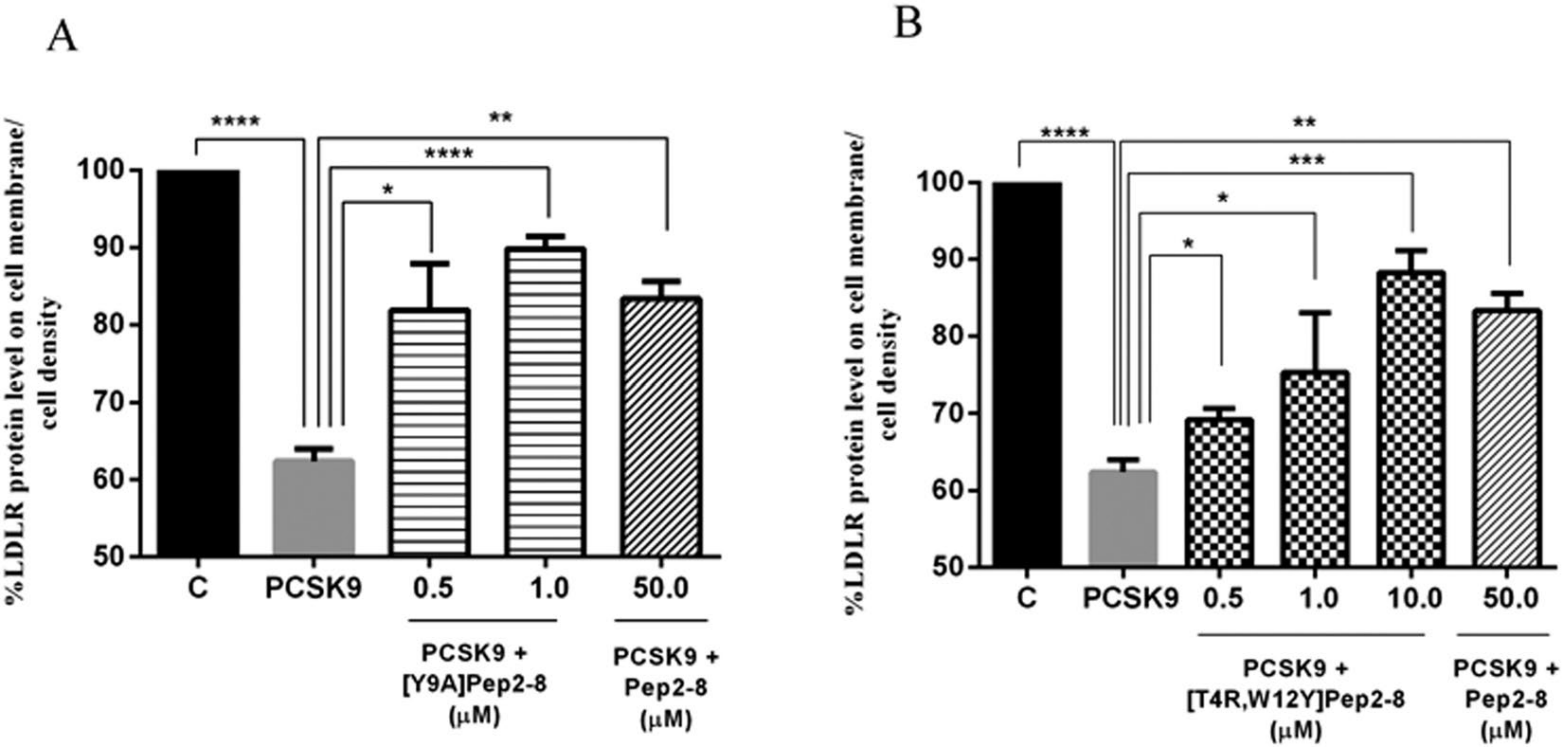
Effect of Pep2-8 derivatives on the LDLR levels. [Y9A]Pep2-8 and [T4R,W12Y]Pep2-8 induce an increase of LDLR protein level on HepG2 cell surface. Briefly, LDLR degradation mediated by PCSK9 is prevented by increasing concentrations of (**A**) [Y9A]Pep2-8 (0.5 and 1.0 µM) and (**B**) [T4R,W12Y]Pep2-8 (0.5–10.0 µM), which was about 50 and 5-fold more potent than the reference peptide (Pep2-8) alone, respectively. C represents control without any treatment. Results are mean ± s.d. of three independent experiments. (*) *P* < 0.05, (**) *P* < 0.001, (***) *P* < 0.0001, and (****) *P* < 0.00001.

Based on the *in vitro* results, the effects of the new peptides on the modulation of the LDLR localized on the HepG2 cell surface were investigated using a new in cell western (ICW) assay^26^. These experiments clearly indicated that in the presence of PCSK9 the LDLR protein levels decreased by 37.62% *versus* the control cells, and that both mutated derivatives can increase the LDLR protein levels when co-incubated with PCSK9. These experiments were performed using Pep2-8 as reference peptide at the fixed concentration of 50 µM. Both peptides gave positive effects. In HepG2 cells incubated with PCSK9, [Y9A]Pep2-8 (0.5 and 1 µM) restored the LDLR protein level up to 81.9% and 89.9%, respectively (Fig. 5A), and [T4R,W12Y]Pep2-8 (0.5, 1.0, and 10.0 µM) up to 68.1%, 75.3%, and 88.3% (Fig. 5B), indicating a concentration-response dependence. On the contrary, Pep2-8 restores the LDLR protein levels up to 83.36% at 50 µM, suggesting that our analogs are more active than the reference peptide.

### By inhibiting PCSK9, engineered peptides improve the ability of HepG2 cell to uptake extracellular LDL

**Figure 6:**
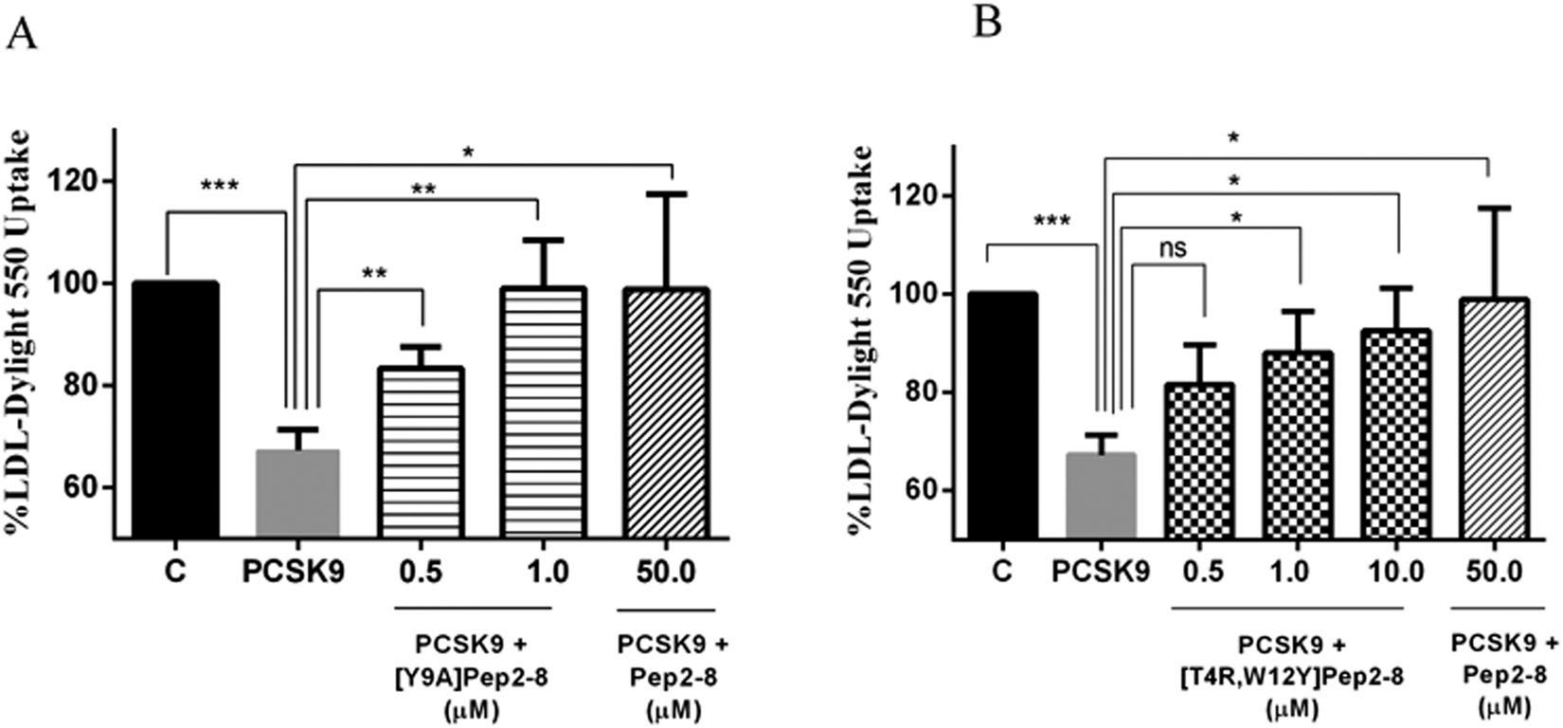
[Y9A]Pep2-8 and [T4R,W12Y]Pep2-8 induce an improved ability of HepG2 cells to uptake LDL from the extracellular environment. Briefly, the decreased ability to uptake LDL by HepG2 cells mediated by PCSK9 is prevented by increasing concentrations of (**A**) [Y9A]Pep2-8 (0.5 and 1.0 µM) and (**B**) [T4R,W12Y]Pep2-8 (0.5–10.0 µM), which was about 50 and 5-fold more potent than the reference peptide (Pep2-8) alone, respectively. C represents control without any treatment. Results are expressed as mean ± s.d. of three independent experiments. (*) *P* < 0.05, (**) *P* < 0.001, and (***) *P* < 0.0001.

To evaluate the ability of the new analogs to modulate the HepG2 cell capacity to uptake extracellular LDL, functional experiments were performed. HepG2 cells were treated with PCSK9 alone or in the presence of [Y9A]Pep2-8, [T4R,W12Y]Pep2-8, or Pep2-8. After 2 h treatment with PCSK9 alone a reduced ability of HepG2 cells to uptake fluorescent LDL by 32.7% *versus* untreated cells was observed (Fig. 6), however this ability was improved by Pep2-8 and each engineered peptide at all tested concentrations. [Y9A]Pep2-8 (0.5 and 1.0 µM) restored the LDL-uptake by 83.4% and 99.0%, respectively, whereas [T4R,W12Y]Pep2-8 (0.5, 1.0, and 10.0 µM) by 81.6%, 88.0, and 92.5%, respectively. Interestingly, the effect produced by 50 µM Pep2-8 (98.9%) was comparable to those induced by [Y9A]Pep2-8 (1.0 µM) and [T4R,W12Y]Pep2-8 (10 µM) indicating that our peptides were about 50 and 5 times more active than Pep2-8 also from a functional point of view.

## Discussion

The inhibition of the PCSK9-LDLR PPI is considered one of the most promising approach to control hypercholesterolemia. In the area of small molecules, phage display-derived peptides, such as Pep2-8^13^, behaving as PCSK9 antagonists have been recently described as well as new Pep2-8 analogs with improved activity on the same biological target and on the LDLR expression on liver cell surface^18^. However, complete studies on the Pep2-8 sequence by alanine-scanning mutational analysis have not been reported yet.

Here, using computational methods, a theoretical prediction of the binding free energy values of the alanine mutated Pep2-8 analogs was accomplished. This study highlights that the residue in position 3 of Pep2-8 is certainly a hotspot of the PCSK9/Pep2-8 interaction, since the mutation into alanine greatly impaired the predicted binding free energy value. On the contrary, peptide [Y9A]Pep2-8 showed a greater theoretical affinity than that predicted for the reference peptide. Our predictions suggested that it is possible to obtain an enhanced complementarity between the C-terminal end of the mutant peptide and the N-terminal grove of PCSK9.

On the other hand, residues in position 4 and 12 do not play any crucial role in the PPI and can be considered preferential sites of Pep2-8 in which new residues could replace the original ones, in order to design new and more potent PCSK9 inhibitors. Then, all amino acids were inserted in those positions and the resulting binding free energy values were calculated. Since the substitution providing the best theoretical affinity was [T4R,W12Y]Pep2-8, this peptide as well as [Y9A]Pep2-8 were synthesized and tested in comparison with the reference peptide Pep2-8. Biochemical experiments, based on a PCSK9-LDLR binding assay, were performed in order to compare the ability of each derivative to impair the PPI between PCSK9 and LDLR. Briefly, the method is based on a colorimetric semi-quantitative solid phase binding assay between recombinant His-tagged PCSK9 protein and recombinant LDLR-AB domain. The color, quantitated by spectrophotometry, reflects the relative amount of recombinant His-tagged PCSK9 protein that binds to recombinant LDLR-AB on microplates. This tool has been optimized and validated in previous studies in which other PCSK9 inhibitors have been developed and characterized^11,12^. Initially, in agreement with recently published conditions^27^, a preliminary screening was performed at the fixed concentration of 100 µM. Figure 4A shows that [Y9A]Pep2-8 and [T4R,W12Y]Pep2-8 were able to decrease the PCSK9 / LDLR PPI much better than Pep2-8. Afterwards, the same biochemical tool was used to measure the IC_50_ values of the PCSK9 / LDLR PPI inhibition that resulted to be equal to 27.12 µM for [Y9A]Pep2-8 and to 14.50 µM for [T4R,W12Y]Pep2-8 (Fig. 4B).

Further experiments were carried out to characterize them from a cellular and functional point of view, choosing the human hepatic HepG2 cell line. In this case, the effects of [Y9A]Pep2-8 and [T4R,W12Y]Pep2-8 were compared with those of Pep2-8 on the modulation of LDL receptor levels on the HepG2 cell surface and the ability of cells to uptake the LDL from extracellular environment.

For the biological characterization, a recently developed ICW assay^26^ was used. This is a colorimetric cell-based assay allowing detection of target proteins in fixed, cultured cells directly in microplates, yielding a higher throughput compared to Western blotting (WB), since it eliminates some typical work-up steps of WB, such as cell lysate preparation, electrophoresis, and membrane transfer^28^. This permits the quick and easy screening of large sample numbers. Moreover, it reduces time and saves money compared to other assays, such as ELISA. ICW assays have generally been used for the selective detection of the phosphorylated states of target proteins using phospho-specific antibodies, in order to investigate phosphorylation pathways and their activators or inhibitors^29^. A specific ICW method has been developed and applied by us to detect the up-regulation of the LDLR directly in the fixed HepG2 cell line to investigate cholesterol metabolism, using a target-specific primary antibody and a horseradish peroxidase (HRP)-conjugated secondary antibody^26^.

This reliable technique was used to investigate the effects of the mutated peptides on the modulation of the LDLR protein levels localized on the HepG2 cell surface. Taking this into consideration, to evaluate only the LDLR surface levels no permeabilization step was included in the applied procedure. More in detail, HepG2 cells were incubated with 4 µg/mL of PCSK9 and in parallel other cells were incubated with PCSK9 in presence of different concentrations of [Y9A]Pep2-8 and [T4R,W12Y]Pep2-8 for 2 h. Figure 5 clearly indicates that the PCSK9 treatment reduced the LDLR levels, which was significantly restored in the presence of the mutated peptides. Interestingly, [Y9A]Pep2-8 and [T4R,W12Y]Pep2-8 were 100 and 50 times more active than Pep2-8 tested at 50 μM.

By using Fluorescence Activated Cell Sorting (FACS) analysis, Zhang and co-workers have demonstrated that, after a 4 h co-incubation with 15 μg/mL PCSK9, Pep2-8 renewed the surface LDLR levels with a concentration-response dependence up to about 70% at 50 μM. This is roughly in agreement with the results obtained by us using the ICW assay (83.4%), suggesting that our ICW assay is reliable and sensitive. It is useful to underline, that in our case the cells were treated with a lower PCSK9 concentration (4 μg/mL) and for a shorter time (2 h) in respect to the conditions applied by Zhang and co-workers.

From a functional point of view, [Y9A]Pep2-8 (0.5 and 1.0 µM) restored the LDL-uptake of PCSK9 treated HepG2 cells up to 83.4% and 99.0%, and [T4R,W12Y]Pep2-8 (0.5, 1.0, and 10.0 µM) up to 81.6%, 88.0, and 92.5%, whereas the reference peptide Pep2-8 restored it up to 98.9% at 50 µM. The value of the reference is in agreement with published data^13^, confirming that the mutated peptides are more active than Pep2-8. It seems thus possible to affirm that indeed the mutated peptides are able to impair the PCSK9 activity, resulting in a functional recovery of PCSK9cellular LDLR.

In a recent study, Zhang and co-workers^18^ have developed new antagonists. In particular, they have found groove-binding peptides using a phage-display strategy and they have fused one of these peptides to the Pep2-8 *via* the GSC linker, obtaining Fusion 1 peptide (TVFTSWEEYLDWV-GSC- CRLPWNLQRIGLPG). Experiments performed on HepG2 cells have demonstrated that the cell surface LDLR degradation mediated by PCSK9 is prevented by increasing concentrations of Fusion 1 peptide, which was about 20-fold more potent than the reference peptide Pep2-8. In particular, in presence of PCSK9, Fusion 1 peptide, tested in a range of concentrations from 0.1 to 5.0 µM, was able to restore the LDLR degradation induced by PCSK9 with a dose-response trend and to restore the LDLR up to 90% at 5.0 µM. Remarkably, our peptide [Y9A]Pep2-8 appeared to be 5-fold more potent than the Fusion 1 peptide, whereas, [T4R,W12Y]Pep2-8 was 2-fold less potent. Based on these evidences, our Pep2-8 analogs seem to possess great potentiality for further successful modifications.

Certainly, we are aware that peptides are not usually considered orally bioavailable, however, they can become valuable starting points for designing new small-molecule drugs, such as peptidomimetics. Moreover, interesting perspectives are currently emerging for PCSK9. New experimental evidences propose an active role of PCSK9 in cancer onset and progression. In this contest, it is important to underline that dyslipidemia and obesity^30^ are often associated to an increased cancer risk and with the diffusion of metastasis^31^. For this reason, lipidogenesis is currently becoming an emerging field for the development of anticancer treatments and PCSK9 an important target for new anticancer treatments.

## Experimental Section

### General Methods

All tested peptides were of analytical grade and were used without further purification. The chemical suppliers were from PRIMM Biotech (Milano, Italy) and GenScript (New Jersey, United States). The peptides were acetylated and amidated on the N-terminus and C-terminus, respectively. For all products, the chemical purity was greater than 95%, as determined by HPLC.

### The PCSK9/Pep2-8 model

Computational studies started retrieving the coordinates of the PCSK9/Pep2-8 complex (PDB code 4NMX, resolution 1.85 Å) from Protein Data Bank (PDB). The unsolved regions of PCSK9 were modeled using the model previously developed by us^11^. Pep2-8, as in the X-ray structure, was acetylated and amidated on the N-terminus and C-terminus, respectively. Furthermore, the rough PCSK9/Pep2-8 model was optimized by energy minimization and MD simulations calculations. In fact, the model was initially solvated with almost 25,000 water molecules (Amber 2017 package)^32^, creating a system composed of more than 80,000 atoms. The FF14SB^33^ version of the AMBER force field was used for the protein, while the TIP3P model^34^ was used to explicitly represent water molecules. Van der Waals and short-range electrostatic interactions were estimated within a 8 Å cutoff, while the long-range electrostatic interactions were included by using the particle mesh Ewald method^35^. Bonds involving hydrogen atoms were constrained using the SHAKE algorithm^36^, enabling the use of a 2 fs time step. After a preliminary minimization run, where only the solvent was optimized, the model was geometrically optimized starting from the side chain conformations and then continuing with the whole protein. Prior to starting the MD simulations, the system was equilibrated for 40 ps at 300 K in isocore conditions (NVT), then isothermal-isobaric ensemble were carried out at 300 K. The Berendsen’s coupling algorithm^37^ was active to maintain the pressure (1 atm) for both solvent and solute molecules, while the coupling constant was set to 1.5 ps. Periodic boundary conditions were applied in the MD simulations and, in the production run, 250 ns of MD simulations were performed by *pmemd.cuda* module of AMBER 2017. Then, the acquired MD trajectory was examined by visual inspection with VMD^38^, ensuring that the thermalization did not cause any structural distortion.

### MM-GBSA calculations

MM-GBSA calculations were performed on the 13 alanine mutants Pep2-8/PCSK9 complexes, built systematically altering the peptide sequence on the PCSK9/Pep2-8 complex previously optimized. The resulting complexes were submitted to energy minimization and MD simulations, adopting the procedure and the parameters described in the previous paragraph. In these cases, more than 100 ns of MD simulations were accomplished in the production runs. These simulations permitted to acquire the trajectories useful for the subsequent binding energy calculations by the “single trajectory” protocol of the MM-GBSA approach^39^. The main advantage of this computational protocol is the use of an ensemble of structures (snapshots) accounting for more than one possible conformation of the protein/peptide complex. Here, snapshots were extracted from each trajectory when the systems reached the geometrical stability, i.e. the Cα atoms RMSF *vs* time plot reached a plateau and the exponential tendency line of the peptide Calpha RMSF did not show any inclination (Fig. S5, Supporting information). Eighty snapshots were regularly extracted to ensure the lowest standard error in the free energy estimation and the lowest calculation time. The time intervals for the extraction were chosen dividing by 80 the number of frames in which the systems showed the geometrical stability. MM-GBSA calculations were performed by *MM-PBSA.py* module^40^ of Amber 2017 package, keeping parameters in the default values. Estimation of entropy contribution is highly demanding from the computational point of view and is has been demonstrated that does not always improve the accuracy for binding free energy predictions^21–24^. “Energy decomposition” calculations were performed by MM-PBSA.py module, to estimate the energy contribution of each residue of PCSK9 when it is in contact with each residue composing the small peptide under investigation (histograms reported in Figs 2 and 3).

### Affinity maturation calculations

Aiming to improve the affinity of Pep2-8, a systematic mutation of the positions 4 and 12 were carried out. The starting PCSK9/Pep2-8 complex was prepared by the ‘protein preparation wizard’ module implemented in *Maestro* and aimed to correctly assign the residue protonation state at pH 7.4, to check the residue completeness and to eliminate atomic clashes. The complex conformation equilibrated by MD simulations was used. Then, all the 400 possible peptides that can derive from the mutation on the two selected positions were generated and the resulting complexes minimized by Prime MM-GBSA, which uses OPLS3^41^ as force field and a continuum solvent models to include the solvent effect into the calculations. Finally, the change in affinity (ΔΔG) between PCSK9 and the generated peptides with respect to Pep2-8 was also estimated by *Prime* MM-GBSA (Prime, Schrödinger, LLC, New York, NY, 2017).

### Materials

The HepG2 cell line was bought from ATCC (HB-8065, ATCC from LGC Standards, Milan, Italy). Dulbecco’s modified Eagle’s medium, (DMEM), 96-Well plates, L-glutamine, fetal bovine serum (FBS), phosphate buffered saline (PBS), penicillin/streptomycin, and chemiluminescent reagent were purchased from Euroclone (Milan, Italy). Janus green were bought from Abcam (Cambridge, UK), while the antibodies against anti-rabbit Ig-HRP, was purchased from Santa Cruz Biotechnology Inc. (Santa Cruz, CA, US). Antibody against LDLR and TMB substrate were obtained from Pierce (Rockford, IL, US). LDL-DyLight™ 550 (Cayman Chemical Company, Ann Arbor, MI, US). Synthetic peptides were synthesized by the company GenScript (Piscataway, NJ, USA) at >95% purity.

### PCSK9-LDLR binding Assay

The synthetic engineered Pep2-8 peptides (1.0 μM–300.0 μM) were tested using the *in vitro* PCSK9-LDLR binding assay (CycLex Co., Nagano, Japan), following the manufacture instructions. The absorbance at 450 nm was measured using the Synergy H1 fluorescent plate reader (Biotek, Bad Friedrichshall, Germany). In particular, for the *in vitro* screening of the synthetic PCSK9-LDLR inhibitors, at different concentrations, were added to the appropriate amount of His-tagged PCSK9 in the wells that had been coated with recombinant LDLR-AB domain, followed by evaluation of inhibitory effect on PCSK9-LDLR interaction by measuring the amount of His-tagged PCSK9 on the wells which is correlated to the absorbance signals at 450 nm, which were measured using the Synergy H1 fluorescent plate reader (Biotek, Bad Friedrichshall, Germany).

### Cell culture conditions

The HepG2 cell line was cultured in DMEM high glucose with stable L-glutamine supplemented with 10% FBS, 100 U/mL penicillin, 100.0 µg/mL streptomycin and incubated at 37 °C under 5% CO_2_ atmosphere. HepG2 cells were used for no more than 20 passages after thawing, because the increase of the number of passages may change the cell characteristics and impair assay results.

### Treatments and conditions

A total of 3.0 × 10^4^ HepG2 cells/well were seeded in 96-well plates, respectively. The following day, cells were washed with PBS and then starved overnight (O/N) in DMEM without FBS. HepG2 cells were treated with 4.0 μg/mL PCSK9-WT (wild type) and 4.0 μg/mL PCSK9 + engineered peptides (0.5–10.0 µM) and/or Pep2-8 (50.0 µM) and vehicle (H_2_O) for 2 h at 37 °C under 5% CO_2_ atmosphere.

### Cell fixation and ICW

Treated HepG2 cells were fixed in 4% paraformaldehyde for 20 min at room temperature (RT). Cells were washed 5 times with 100.0 µL of PBS/well (each wash was for 5 min at RT) and the endogenous peroxides activity was quenched adding 3% H_2_O_2_ in PBS for 20 min at RT. Non-specific sites were blocked with 100.0 µL/well of 5% BSA in PBS for 1.5 h at RT. LDLR primary antibody solution (1:3000 in 5% BSA in PBS, 25 µL/well) was incubated O/N at 4 °C. Subsequently, the primary antibody solution was discarded and each sample was washed 5 times with 100.0 µL/well of PBS (each wash was for 5 min at RT). Goat anti-rabbit Ig-HRP secondary antibody solution (1:6000 in 5% BSA in PBS, 50.0 µL/well) was added and incubated for 1 h at RT. The secondary antibody solution was washed 5 times with 100.0 µL/well of PBS (each wash for 5 min at RT). Freshly prepared TMB substrate (100.0 µL/well), was added and the plate was incubated at room temperature (RT) until desired color was developed. The reaction was then stopped with 2 M H_2_SO_4_ and the absorbance at 450 nm was measured using the Synergy H1 fluorescent plate reader from Biotek. Cells were stained by adding 1 × Janus green stain, incubating for 5 min at RT. The dye was removed and the sample washed 5 times with water. Afterward 0.1 mL 0.5 M HCl per well were added and incubated for 10 min. After 10 sec shaking, the OD at 595 nm was measured using the Synergy H1 fluorescent plate reader from Biotek.

### Fluorescent LDL uptake cell based assay

HepG2 cells (3.0 × 10^4^/well) were seeded in black 96-well plates and kept in complete growth medium for 2 d before treatment. The third day, they were treated with 4.0 μg/mL PCSK9 and 4.0 μg/mL PCSK9 + peptides (0.5-50.0 µM), and vehicle (H_2_O) for 2 h with at 37 °C under 5% CO_2_ atmosphere. At the end of the treatments, the culture medium was replaced with 50.0 μl/well LDL-DyLight™ 550 working solution (Cayman Chemical Company, Ann Arbor, MI, US). The cells were additionally incubated for 2 h at 37 °C and then the culture medium was aspirated and replaced with PBS (100.0 μl/well). The degree of LDL uptake was measured using the Synergy H1 fluorescent plate reader from Biotek (excitation and emission wavelengths 540 and 570 nm, respectively).

### Statistical analysis of biological assays

Data are presented as mean ± s.d. using GraphPad Prism 6 (GraphPad, La Jolla, CA, USA). Statistical analyses were carried out by *t* student test. P-values < 0.05 were considered to be significant.

## Electronic supplementary material

Supporting Information
