# Supplementary material for: Biological Characterization of Computationally Designed Analogs of peptide TVFTSWEEYLDWV (Pep2-8) with Increased PCSK9 Antagonistic Activity
Source: Sci Rep. 2019 Feb 20;9:2343. doi: 10.1038/s41598-018-35819-0 (PMC6382862; doi:10.1038/s41598-018-35819-0)
Supplement: Supplementary file 1 — Supporting Information [file 41598_2018_35819_MOESM1_ESM.pdf]

SUPPORTING INFORMATION

Biological Characterization of Computationally Designed Analogs of peptide TVFTSWEEYLDWV (Pep2-8) with Increased PCSK9 Antagonistic Activity

Carmen Lammi,[a] Jacopo Sgrignani,[b] Anna Arnoldi,[a] Giovanni Grazioso,[a]*

[a] Dipartimento di Scienze Farmaceutiche, Università degli Studi di Milano, Via L. Mangiagalli 25, 20133 Milan, Italy.

[b] Istituto di ricerca in biomedicine (IRB), Università della Svizzera Italiana (USI), Via V. Vela 6, CH-6500, Bellinzona, Switzerland.

Contents

MD Simulations Outcomes ………………………………………………………….……S2

Figure S4 ……………………………………………………………………………….....S6

References ………………………………………………………………………………...S6

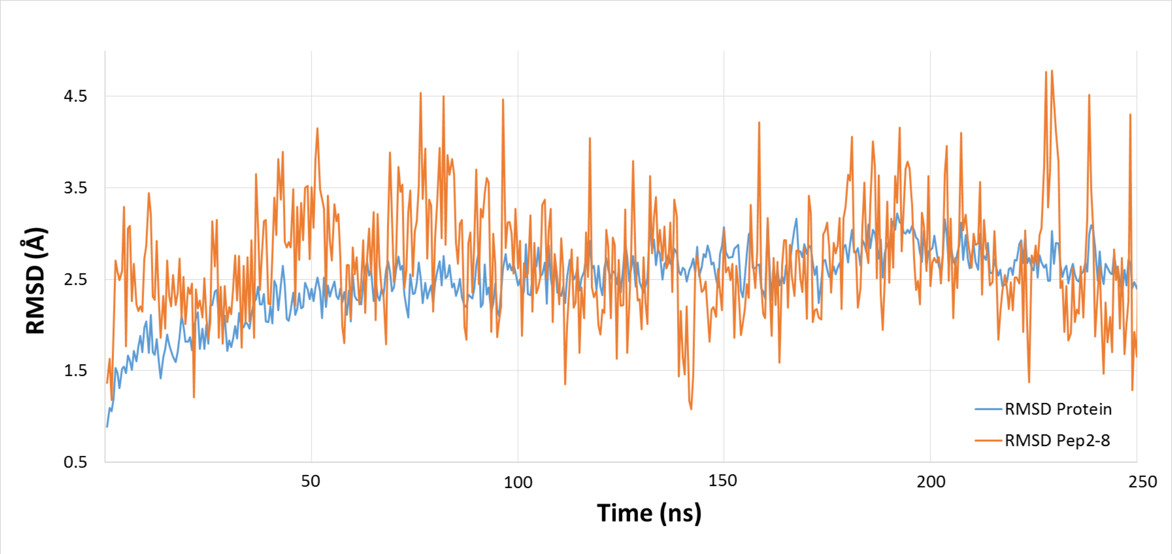

Figure S1. RMSD values as a function of simulation time for a 250 ns long MD simulations on PCSK9/Pep2-8 complex (cyan line). The orange line highlights the RMSD values of Pep2-8. The complex coordinate attained after preliminary equilibration process was used as reference structure for RMSD calculations.

MD simulations outcomes. As described in the main text, each residue composing Pep2-8 has been carefully examined in the light of: i) the interactions produced with PCSK9 in the X-ray crystal structure; ii) the stability of those interactions over MD simulations, and iii) the effects produced by the alanine mutation on the peptide ΔG* values. Details are reported in the main text and in the following sections.

T1. The X-ray structure showed that a water molecule bridges the carbonyl group of T1 to the side chain of PCSK9-S381, while the side chain of T1 creates an internal van der Waals (vdW) contact with the side chain of Pep2-8F3. MD simulations on the template peptide suggest that the hydrogen bond (Hb) network shaped by water molecule is fleeting since it was rapidly disrupted in the early stages of MD simulations. The mutation into alanine of T1 did not significantly increase any interaction of the peptide with the PCSK9 surface. The theoretical binding free energy value of [T1A]Pep2-8 was not significantly higher than that of Pep2-8 (+1.2 kcal/mol, Table 1 in the main text).

V2. The PCSK9/Pep2-8 crystal structure suggests that the backbone of Pep2-8V2 is bound, by hydrogen bonds (Hbs), to one of PCSK9-F379, while the isopropyl group of V2 is in contact with the side chain of PCSK9-D734. Over MD simulations, the side chain of V2 created vdW contacts with the side chain of PCSK9-V380. The mutation of V2 into alanine did not cause any large changes in the secondary structure of the peptide, as well as on the β-strand shaping the N-terminal end of Pep2-8. The binding free energy of the mutant peptide was slightly higher (-31.7 kcal/mol) than Pep2-8. In literature, the mutation V2A led to a peptide with lower affinity than Pep2-8, although still detectable by SPR.[1]

F3. In the X-ray, the backbone of Pep2-8F3 is bound by a Hb with the NH group of PCSK9-F379. This residue is the first amino acid of the β-strand, creating the β-sheet with the PCSK9 residues from C378 to S381. In the X-ray, the benzyl ring of F3 is packed with the side chains of Pep2-8W12 and Pep2-8Y9, and this interaction was stable over MD simulations. The mutation of Pep2-8F3 into alanine led to low conformational stability of the peptide on PCSK9. In fact, due to the lack of the phenyl ring into the *core* of the peptide, the N-term cap showed higher conformational freedom and, at the end of simulations, the cap (acetyl group) reached the area of Pep2-8 previously occupied by the side chains of the side chains of Pep2-8W12 and Pep2-8Y9. The central role of Pep2-8F3 in the binding of Pep2-8 on PCSK9 is additionally proved by calculating the binding free energy of the

mutant peptide. In fact, MM-GBSA calculations suggested that [F3A]Pep2-8 has a theoretical affinity 7.1 kcal/mol (Tables 1, main text) lower than Pep2-8.

T4. In the X-ray structure this residue, bound by the NH group and side chain to the carboxyl group of Pep2-8E8, creates an additional Hb with the side chain of PCSK9-T377, using the backbone oxygen atom. Although all Hbs were not completely stable over MD simulations, they were all retained over MD production runs.

S5. S5 constitutes the N-terminal residue of the Pep2-8 α-helix. As in the canonical α-helix, the carbonyl group of the residue *i* creates a Hb with the NH group of the residue *i+4*, as has been found between residues Pep2-8S5 and Pep2-8Y9 in the X-ray structure. An additional Hb is created between the side chain of Pep2-8S5 and the NH group of Pep2-8E8. Interestingly, these interactions remained unbroken over the MD simulations. The secondary structure of [S5A]Pep2-8 peptide did not show any substantial changes, in the secondary structure, as well as in the contacts with PCSK9. It is remarkable that the lack of the hydroxyl group in position five on the mutant peptide leaves the side chain of Pep2-8E8 free to fluctuate on the PCSK9 surface, decreasing the conformational stability of the peptide on PCSK9.

W6. This residue, located in the α-helix, creates a Hb by the carbonyl group with the NH of Pep2-8E10. The indole ring is projected cavity sized by PCSK9-D238, PCSK9-F379, PCSK9-I369, PCSK9-P155 and the phenol ring of Pep2-8Y9. All of this contacts were stable over MD simulations but, interestingly, the pocket previously filled by the indole ring was than occupied by the side chain of Pep2-8Y9 of the mutant peptide, shaping a π-π contact with the benzyl ring of PCSK9-F379. Consequently, at the end of MD simulations, the α-helix of the mutant peptide was shifted in the direction of PCSK9-T377 and the PCSK9-C375_C378 disulfide bridge. MM-GBSA calculations confirm these outcomes. In fact, the mutant peptide showed ΔG* value higher than Pep2-8.

E7. In the X-ray structure, the NH group of E7 creates a Hb with its side chain while, the carbonyl group, stabilizes the α-helix creating a Hb with the NH of Pep2-8D11. MD simulations did not significantly alter the binding mode of Pep2-8. Moreover, the Pep2-8E7 side chain frequently created electrostatic interaction with a positively charged area shaped by PCSK9-R194 and -R237. The mutation of Pep2-8E7 into alanine did not significantly modify the binding mode of the peptide.

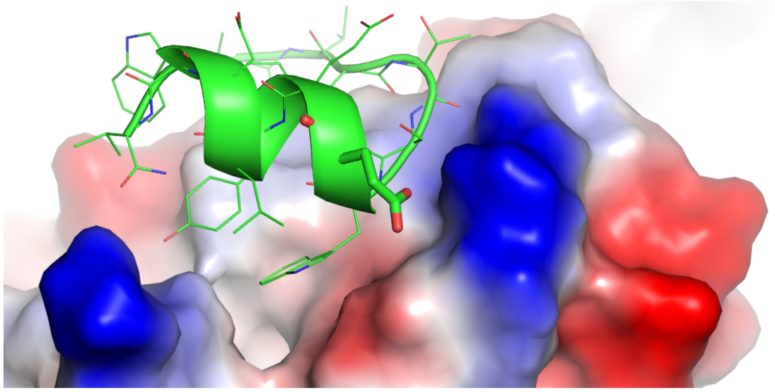

Figure S2. Binding mode of Pep2-8 on the PSCK9 surface. Pep2-8E7 residues has been depicted as green sticks. The remaining Pep2-8 residues are shown as thin sticks. On the PCSK9 solvent accessible surface has been project the partial charges of the PCSK9 surrounded by solvent: blue for positive charges, white for neutral and red for negative charges. Picture was acquired by pymol software.

E8. This residue is located on the external surface of the complex, surrounded by solvent molecules. The side chain of Pep2-8E8 creates two Hbs with Pep2-8T4, whereas the carbonyl group stabilizes the α-helix by a Hb with the NH group of Pep2-8W12. The Hb between the Pep2-8T4 and Pep2-8E8 side chains was constantly established over MD simulations. Nevertheless, the secondary structure of the mutant peptide was not stable during MD simulations. In fact, the α-helix shaped by residues W6-W12, in the [E8A]Pep2-8 was created by T4 and E8. Residues from D11 to V13 created a turn, whereas the N-terminal β-strand appeared as random coil. The ΔG* values of [E8A]Pep2-8 was similar to the one of Pep2-8, though a dramatic change in the secondary structure of the peptide was triggered by the E8A mutation.

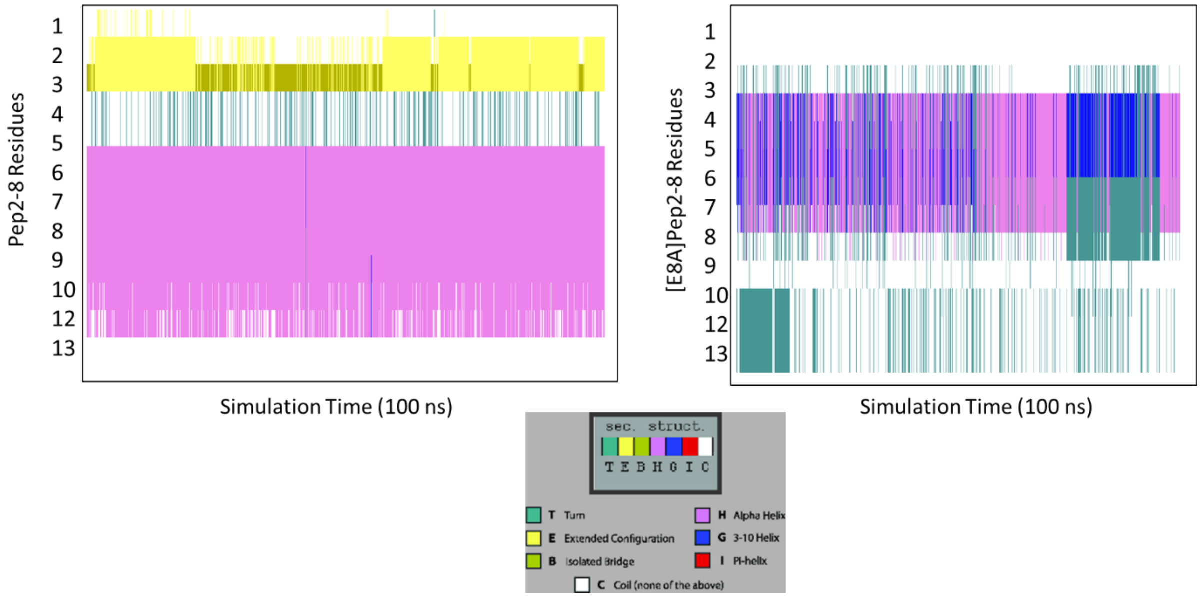

Figure S3. Timeline analysis showing alterations in the small peptide secondary structure, over MD simulations of the PCSK9 complexes. "X" axis represents time period and "Y" axis represents changes in secondary structure of Pep2-8 (left panel) and [E8A]Pep2-8 (panel on the right). For PCSK9/Pep2-8 complex 250 ns-long MD simulations were initially performed. In order to compare the results between the "wild" and the mutated ligand, only the first 100 ns of the PCSK9/Pep2-8 MD simulations were considered. Color key: the symbol 'T' (cyan) represents hydrogen bonded turn, 'E' (yellow) r extended β-sheet in parallel or anti-parallel, 'B' (green) single pair β-bridge, the helices 'H' (magenta), 'G' (blue) and 'I' (red) 4, 3, 5-turn helix and 'C' (white) s coil.

Y9. In the PCSK9/Pep2-8 crystal structure, the side chain Pep2-8Y9 is inserted in a pocket of PCSK9 shaped by P155, I369 and the side chain of Pep2-8W6. Interestingly, the phenol group of Pep2-8Y9 did not create any Hb with the biological counterpart. The CO group of Pep2-8Y9 created a Hb with the Pep2-8V13 amide, while the NH created a Hb with the carbonyl group of Pep2-8S5. All these contacts were retained over MD simulations, although the mutant peptide reserved unexpected results (see main text for details).

L10. The X-ray crystal structure of Pep2-8 within PCSK9 state that the carbonyl group of Pep2-8W6 creates a Hb with NH atoms of Pep2-8L10. Both residues were in close contact also using their side chains. These interactions were stable over MD simulations and a Hb, between the carbonyl group of this residue and amide group at the C-terminal end, was occasionally created. MD simulations on [L10A]Pep2-8 did not show any substantial changes in the Hb network, as well as in the secondary structure of the small peptide. It seems that the side chain of Pep2-8L10 does not play a crucial role in the Pep2-8/PCSK9 PPI, being located on the external surface of the complex, surrounded by solvent.

D11. Crystallographic data suggest that this residue does not play a role diverse from the previous one. Projecting the side chain in the solvent in the solvent, Pep2-8D11 cannot be considered a hotspot of Pep2-8. Accordingly, the [D11A]Pep2-8 mutant did not show altered contacts with PCSK9.

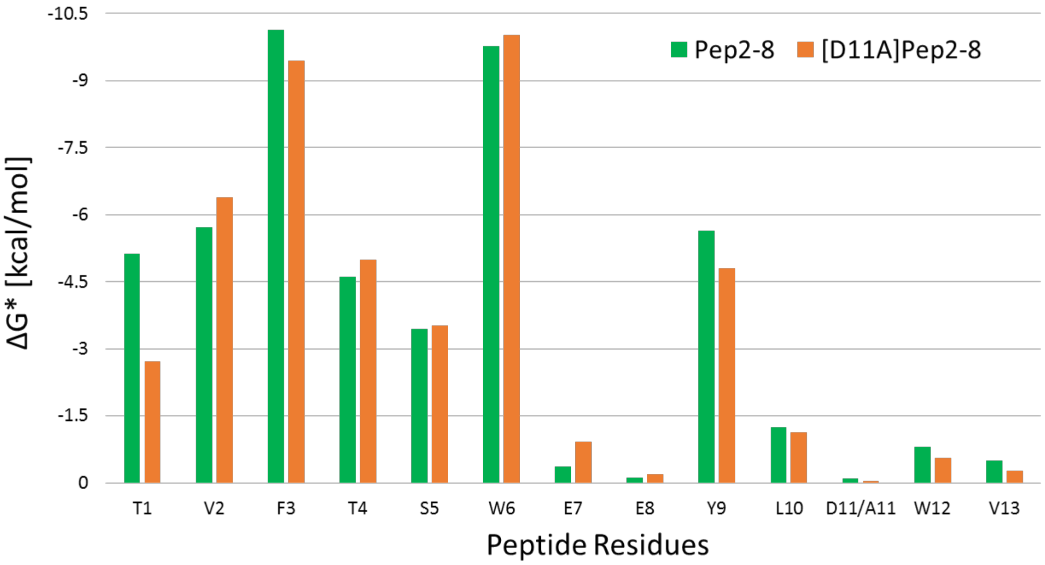

Figure S4. Histogram showing the calculated per-residue contributions to the free energy calculated using MMGBSA approach.

W12. As reported above, the NH group of Pep2-8W12 creates, as seen in the X-ray structure, a Hb with the backbone of Pep2-8E8. In turn, the indole ring of W12 creates vdW contacts with the side chains of Pep2-8F3 and Pep2-8V13. MD simulations on the solvated crystal structure did not evidence large variations in the Pep2-8/PCSK9 PPIs. Similarly, the W12A mutation, and further MD simulations, did not show any alterations of the peptide binding mode. Nevertheless, it is interesting to note the side chain of W12 is in close contact with PCSK9.

V13. As resulted by the X-ray studies, this residue is near Pep2-8Y9. Moreover, this residue, constituting the C-terminal end of Pep2-8, created a Hb with the hydroxyl group of PCSK9-S153, by its amide. Unfortunately, the high conformational freedom of the PCSK9 N-terminal ends, makes this interaction unstable. In the [V13A]Pep2-8 peptide the creation of a Hb between the C-terminal cap and the carbonyl group of Pep2-8D11 was noted. A Hb between the amino group of PCSK9-S153 and the carbonyl group of Pep2-8A13 additionally stabilize the peptide in the PCSK9 binding site.

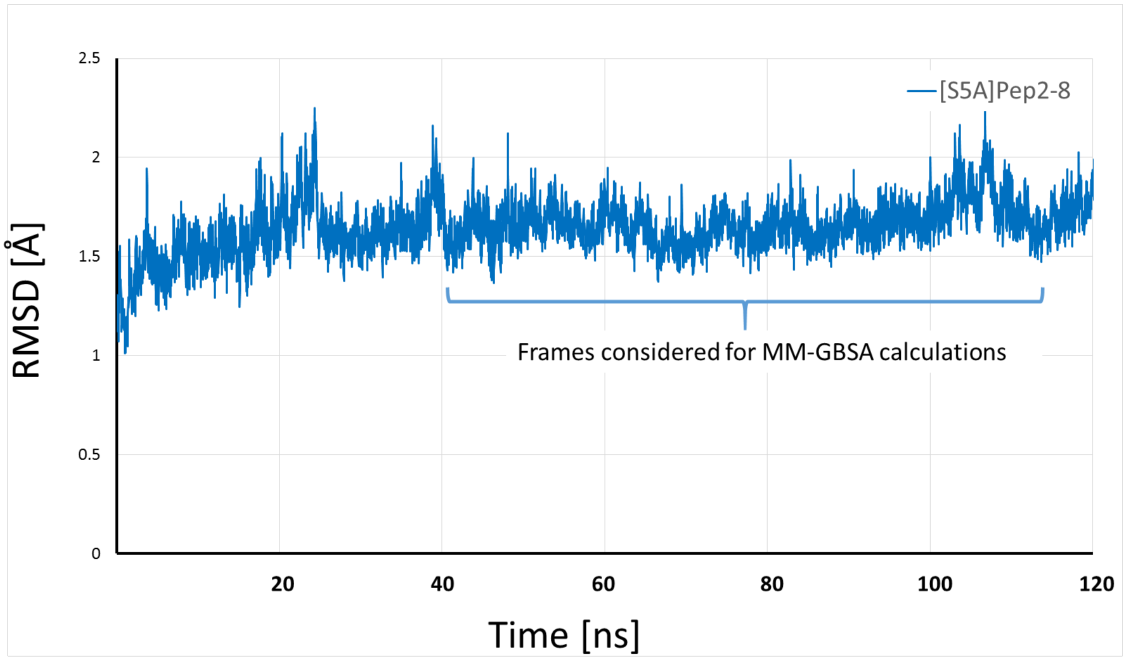

Figure S5. RMSD (Å) values of small peptide over simulation time of the complex PCSK9/[S5A]Pep2-8 Cα atoms aligned on the equilibrated structure. The frames considered for the MM-GBSA calculations are also shown in the plot.

References

1. Zhang, Y., Ultsch, M., Skelton, N. J., Burdick, D. J., Beresini, M. H., Li, W., Kong-Beltran, M., Peterson, A., Quinn, J., Chiu, C., Wu, Y., Shia, S., Moran, P., Di Lello, P., Eigenbrot, C., Kirchhofer, D. (2017) Discovery of a cryptic peptide-binding site on PCSK9 and design of antagonists. *Nat Struct Mol Biol* **24**, 848-856.
